# Effectiveness of Digital Education on Communication Skills Among Medical Students: Systematic Review and Meta-Analysis by the Digital Health Education Collaboration

**DOI:** 10.2196/12967

**Published:** 2019-08-27

**Authors:** Bhone Myint Kyaw, Pawel Posadzki, Sophie Paddock, Josip Car, James Campbell, Lorainne Tudor Car

**Affiliations:** 1 Centre for Population Health Sciences Lee Kong Chian School of Medicine Nanyang Technological University Singapore Singapore; 2 Norfolk & Norwich University Hospital Colney Lane, Norwich United Kingdom; 3 Health Workforce Department, World Health Organization Geneva Switzerland; 4 Family Medicine and Primary Care Lee Kong Chian School of Medicine Nanyang Technological University Singapore Singapore; 5 Department of Primary Care and Public Health School of Public Health Imperial College London London United Kingdom

**Keywords:** randomized controlled trials, effectiveness, systematic review, communication skills, medical education

## Abstract

**Background:**

Effective communication skills are essential in diagnosis and treatment processes and in building the doctor-patient relationship.

**Objective:**

Our aim was to evaluate the effectiveness of digital education in medical students for communication skills development. Broadly, we assessed whether digital education could improve the quality of future doctors’ communication skills.

**Methods:**

We performed a systematic review and searched seven electronic databases and two trial registries for randomized controlled trials (RCTs) and cluster RCTs (cRCTs) published between January 1990 and September 2018. Two reviewers independently screened the citations, extracted data from the included studies, and assessed the risk of bias. We also assessed the quality of evidence using the Grading of Recommendations, Assessment, Development, and Evaluations assessment (GRADE).

**Results:**

We included 12 studies with 2101 medical students, of which 10 were RCTs and two were cRCTs. The digital education included online modules, virtual patient simulations, and video-assisted oral feedback. The control groups included didactic lectures, oral feedback, standard curriculum, role play, and no intervention as well as less interactive forms of digital education. The overall risk of bias was high, and the quality of evidence ranged from moderate to very low. For skills outcome, meta-analysis of three studies comparing digital education to traditional learning showed no statistically significant difference in postintervention skills scores between the groups (standardized mean difference [SMD]=–0.19; 95% CI –0.9 to 0.52; *I*^2^=86%, N=3 studies [304 students]; small effect size; low-quality evidence). Similarly, a meta-analysis of four studies comparing the effectiveness of blended digital education (ie, online or offline digital education plus traditional learning) and traditional learning showed no statistically significant difference in postintervention skills between the groups (SMD=0.15; 95% CI –0.26 to 0.56; *I*^2^=86%; N=4 studies [762 students]; small effect size; low-quality evidence). The additional meta-analysis of four studies comparing more interactive and less interactive forms of digital education also showed little or no difference in postintervention skills scores between the two groups (SMD=0.12; 95% CI: –0.09 to 0.33; *I*^2^=40%; N=4 studies [893 students]; small effect size; moderate-quality evidence). For knowledge outcome, two studies comparing the effectiveness of blended online digital education and traditional learning reported no difference in postintervention knowledge scores between the groups (SMD=0.18; 95% CI: –0.2 to 0.55; *I*^2^=61%; N=2 studies [292 students]; small effect size; low-quality evidence). The findings on attitudes, satisfaction, and patient-related outcomes were limited or mixed. None of the included studies reported adverse outcomes or economic evaluation of the interventions.

**Conclusions:**

We found low-quality evidence showing that digital education is as effective as traditional learning in medical students’ communication skills training. Blended digital education seems to be at least as effective as and potentially more effective than traditional learning for communication skills and knowledge. We also found no difference in postintervention skills between more and less interactive forms of digital education. There is a need for further research to evaluate the effectiveness of other forms of digital education such as virtual reality, serious gaming, and mobile learning on medical students’ attitude, satisfaction, and patient-related outcomes as well as the adverse effects and cost-effectiveness of digital education.

## Introduction

Both qualitative and quantitative researchers have intensely studied the importance of communication between patients and doctors since the 1970s. Within health care, where an individual explores the unknown environment of one’s own health and disease, effective communication skills can positively affect a number of health outcomes including better emotional and physical health, higher symptom resolution, improved pain control, greater treatment compliance, and enhanced patient satisfaction [1]. Furthermore, studies have reported reductions in emotional distress, levels of discomfort, concerns, fear, hopelessness, grief, depression, or health services utilization as a result of effective communication [2,3]. Communication involves respecting the persons’ dignity, integrity, and autonomy [4,5] as well as an ability to explore and discuss their expectations or wishes in a warm, nonjudgmental, and friendly manner. Effective communication (verbal and nonverbal) includes traits such as empathy, understanding, active listening, and the ability to meet patients’ needs and emotionally charged information [6]. In clinical practice, effective communication also requires features needed for effective symptom control such as honesty, open disclosure, an ability to gain trust [7], and influence over patient behavior [8]. These communication skills are essential in building the doctor-patient relationship or “therapeutic alliance.” Finally, physicians have legal, ethical, and moral obligations to demonstrate a variety of communication skills including the ability to gather information, formulate an accurate diagnosis, provide therapeutic instructions and medical advice, communicate risk, and deliver health-related news to the patients [9,10].

Communication skills training is recognized as an important component of the curricula in undergraduate and postgraduate medical education and is endorsed, for example, by the UK General Medical Council, which states that students should be able to “communicate clearly, sensitively and effectively with patients, their relatives and colleagues” [11]. The optimal method of teaching and learning communication skills is considered a direct observation of the student’s performance, followed by feedback from an experienced tutor [12,13]. This form of small-group teaching requires intensive planning and resources including simulated patients and experienced tutors. The lack of standardization within these patients and tutors can result in unequal learning outcomes.

Digital education encompasses a broad spectrum of didactic interventions characterized by their technological content, learning objectives/outcomes, measurement tools, learning approaches, and delivery settings. Digital education includes online digital education, offline digital education, massive open online courses, learning management systems, mobile digital education (mobile learning or m-learning), serious games and gamification, augmented reality, virtual reality, or virtual patient (VP) [14-17].

For medical students learning communication skills, digital education offers self-directed, flexible, and interactive learning (didactic); novel instructional methods; and the ability to simulate and rehearse different clinical scenarios (experiential learning) [18]. For instance, online digital education could be a potential method of delivering the theoretical concepts that underpin communication skills. Virtual patient simulations may also be useful in clinical scenarios that are difficult to replicate with standardized patients, such as communication with patients who have rare conditions, speech disorders, and neurological diseases. Digital education can be utilized flexibly and for an unlimited number of times alongside traditional methods such as role play with standardized patients, allowing students to practice their skills “interchangeably.” For educators, digital education offers the potential to free up time, save manpower and space resources, automate evaluation and documentation of students’ progress, and receive feedback from the students [19].

Given the shortage of trained and experienced health care educators to deliver communication skills training, digital education may be a novel, cost-effective modality. To the best of our knowledge, there is no similar systematic review assessing the effectiveness of digital education for medical students’ communication skills training. The aim of this research was to evaluate the effectiveness of digital education compared with various controls in improving knowledge, skills, attitudes, and satisfaction of medical students learning communication skills. In doing so, we aim to fill an important gap in the literature.

## Methods

For this systematic review, we adhered to the Preferred Reporting Items for Systematic Reviews and Meta-Analyses Guidelines and the Cochrane Handbook for Systematic Reviews of Interventions [20]. For a detailed description of the methodology, please refer to the study by Car et al [21].

### Eligibility Criteria

We considered studies eligible for inclusion if they were randomized controlled trials (RCTs) of any design and of any type of digital education including blended education (combination of digital education and traditional learning) for medical students (ie, preregistration); measuring any of the primary outcomes, ie, knowledge, skills, attitudes, satisfaction; or measuring secondary outcomes, ie, patient-related outcomes, adverse effects, or costs (economic evaluations). We included studies if the studies compared: digital education versus traditional learning, digital education versus other forms of digital education, digital education versus no intervention, blended digital education versus traditional learning, and blended digital education versus no intervention.

We did not exclude participants based on age, gender, or any other sociodemographic factor. If data within a study included both preregistration (undergraduate level) and postregistration (postgraduate level) students, the study was included if these data were presented separately. We did not impose any language restrictions. Nonrandomized studies or trials of postgraduates including continuous professional development; continuous medical education; and students of traditional, alternative, and complementary medicine were excluded.

### Search Strategy and Data Sources

We searched the following databases from January 1, 1990, to September 20, 2018, for all relevant digital education trials: Cochrane Central Register of Controlled Trials (Wiley), Educational Resource Information Centre (Ovid), Embase (Elsevier), Cumulative Index to Nursing and Allied Health Literature (Ebsco), MEDLINE (Ovid), PsychINFO (Ovid), and Web of Science Core Collection. We also searched the two trials registers—International Clinical Trials Registry Platform and metaRegister of Controlled Trials—to identify unpublished trials. We selected 1990 as the starting year for our search because the use of computers was limited to very basic tasks prior to this year. There were no language restrictions. We searched reference lists of all the studies that we deemed eligible for inclusion in our review and relevant systematic reviews. For a detailed search strategy for MEDLINE, please see Multimedia Appendix 1.

### Data Selection, Extraction, and Management

We merged the search results from the databases using EndNote software [computer software] (Version X.7.8. Philadelphia, PA: Clarivate Analytics) and removed duplicates of the same record. Three reviewers (PP, SP, and BK) independently screened titles and abstracts to identify potentially eligible articles. They then read the full-text versions of these studies and assessed them independently against the inclusion and exclusion criteria. Any disagreements about whether a study meets the eligibility criteria were resolved through discussion among the two review authors. A third review author’s opinion was sought to resolve any disagreements between two review authors. If a study had more than one intervention group, for comparison, we chose the relevant digital education group (ie, more interactive intervention) against the least interactive controls. “Interactivity” referred to “the degree of control or adaptiveness a user might have with a system, without necessarily having to give a response” [22], and we applied this definition of “interactivity” throughout the review. For each of the included studies, two reviewers independently extracted data related to the characteristics of population, intervention, comparators, outcome measures, and study design, and any discrepant opinions were resolved by discussion.

### Assessment of Risk of Bias

Three review authors (PP, SP, and BK) independently assessed the risk of bias of the included studies using the Cochrane Risk of Bias Tool [20]. Disagreements between the reviewers were resolved by discussion. We appraised the following domains: random sequence generation, allocation concealment, blinding (participants, personnel and outcome assessors), completeness of outcome data, selective outcome reporting, and other biases. Each item was judged as having high, low, or unclear risk of bias based on the definitions provided by Higgins and Green [20]. For cluster RCTs, the risk of bias assessment also focused on recruitment bias, baselines imbalance, loss of clusters, incorrect analysis, and comparability with individually randomized controlled trials [23]. We incorporated the results of the risk of bias assessment into the review using a graph and a narrative summary.

### Data Synthesis and Analysis

For continuous outcomes, we reported postintervention mean scores and SD along with the number of participants in each intervention group. We reported postintervention mean outcome data to ensure consistency across the included studies, as most of the included studies (92%) reported postintervention data. We presented outcomes using postintervention standardized mean difference (SMD) and interpreted the effect size based on the Cohen rule of thumb (ie, with 0.2 representing a small effect, 0.5 representing a moderate effect, and 0.8 representing a large effect) [20,24]. If studies had multiple arms, we compared the most interactive intervention arm to the least interactive control arm and assessed the difference in postintervention outcomes.

For dichotomous outcomes, we summarized relative risks and associated 95% CIs across studies. Subgroup analyses were not feasible due to the limited number of studies within respective comparisons and outcomes. We used a random-effects model for meta-analysis. We used the *I*^2^ statistic to evaluate heterogeneity, with *I*^2^ <25%, 25%–75%, and >75% representing low, moderate, and high degree of inconsistency, respectively. The meta-analysis was performed using Review Manager 5.3 [25]. We reported the findings in line with the PRISMA reporting standards [26].

The three authors (SP, PP, and BK) independently assessed the overall quality of the evidence in accordance with the Grading of Recommendations, Assessment, Development and Evaluations criteria [27]. The following criteria were considered: limitations of studies (risk of bias), inconsistency, indirectness, imprecision and publication bias, and downgrading the quality where appropriate. We did this for all primary and secondary outcomes reported in the review. We rated the quality of evidence for each outcome as “high,” “moderate,” and “low.” We prepared “Summary of findings” tables for each comparison to present the findings and rated the quality of the evidence for each outcome (Multimedia Appendices 2-4). We were unable to pool the data statistically using meta-analysis for some outcomes (eg, attitude and satisfaction) due to high heterogeneity in the types of participants, interventions, comparisons, outcomes, outcome measures, and outcomes measurement instruments. We presented those findings in the form of a narrative synthesis. We used the standard method recommended by Higgins et al [20] to synthesize and represent the results.

## Results

### Overview

**Figure 1:**
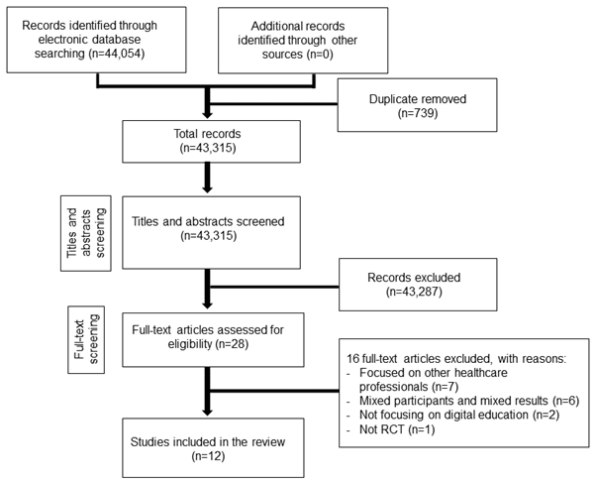
Preferred Reporting Items for Systematic Reviews and Meta-Analyses flow diagram. RCT: randomized controlled trial.

We identified 44,054 records overall from electronic database searches. We excluded 43,287 references after screening titles and abstracts and retrieved 28 studies for full-text evaluation, of which 12 studies met the inclusion criteria [28-39] and were included in the review (Figure 1). The total number of students was 2101.

We present details of the included trials in Table 1. The included studies were published between 2000 and 2018; of these, nine were RCTs, two were cluster RCTs [31,38], and one was a factorial-design RCT [30]. The studies originated from Australia [28], China [39], Germany [30,37], and the United States [29,31-36,38]. The sample sizes in the included studies ranged from 67 to 421 medical students, and they were in their first, second, third, and fourth year of studies. The included studies focused on different modalities of digital education. For instance, five studies (41.7%) [28,33,35,36,38] used VP, whereas the remaining seven studies (58.3%) used online modules; in addition, five studies (41.7%) used traditional learning in addition to digital education, that is, blended digital education [30,31,34,37,39]. Two studies (22.2%) had more than one intervention arm [29,30]. The content of those interventions also differed from history-taking and basic communication skills [28,30,33,36,37], cross-cultural communication [32], ethical reasoning [34], suicide risk management [35], interprofessional communication [38], ophthalmology-related communication skills training [39], and substance abuse–related communication [31] to end-of-life support [29]. Comparison groups ranged from other digital education such as virtual patient [28], online learning [38], traditional learning (written curriculum, didactic lecture, oral feedback, and standardized patient) [29,31-34,36,37,39], video group [35], or no intervention [30]. Outcomes were measured using a range of tools including scales, surveys, checklists, Likert scales, and Objective Structured Clinical Examination (OSCE), questionnaires; seven studies (58.3%) reported some type of validity evidence (ie, validity, reliability, and responsiveness for those measurement tools) [28,30,31,33-35,39].

**Table 1:** Main characteristics of the included studies (N=12).

Study author names, year, country, and design		Number of participants (year)	Intervention and comparator	Outcome/measurement instrument (validity, reliability)	Results (postintervention): SMD^a^ (95% CI)	Effect estimate
**Digital education versus traditional learning**						
	Chittenden et al, 2013 [29] US, RCT^b^	121 (third)	IG1^c^: 45-min online module (multimedia) CG^d^: Traditional learning (written curriculum)	Skills: checklist, Likert scale	Skills: SMD=0.00 (–0.46 to 0.46)	Skills: Digital education=traditional learning
	Deladisma et al, 2007 [33] US, RCT	84 (second)	IG: VP^e^ simulation CG: Traditional learning (SP^f^ consultation)	Skills: survey, Likert scale (Cronbach alpha=.87)	Skills: SMD=–0.89 (–1.35, –0.43)	Skills: Digital education<traditional learning
	Gartmeier et al, 2015 [30] Germany, factorial RCT	168 (second and third)	IG1: 300-min online tutorial CG1: No intervention (wait list)	Skills: Likert scale (intraclass correlation=0.54)	Narrative presentation	Skills: Digital education>traditional learning
	Kaltman et al, 2018 [36] US, RCT	99 (first)	IG: VP simulation (video-based) CG: Traditional learning (usual curriculum)	Skills: OSCE^g^	Skills: SMD=0.33 (–0.16 to 0.81)	Skills: Digital education=traditional learning
**Blended digital education versus traditional learning**						
	Fleetwood et al, 2000 [34] US, RCT	173 (second)	IG: 45-min online module plus small group discussions (blended digital education) CG: 45-min traditional learning (group discussions)	Knowledge: MCQ^h^ (exam) Skills: checklist (validated) Patient-related outcome (ie, patients’ satisfaction): checklist (validated)	Knowledge: SMD=0.00 (–0.30 to 0.30) Skills: SMD=–0.20 (–0.50 to 0.10) Patients’ satisfaction: SMD=–0.43 (–0.73 to –0.13)	Knowledge: Blended digital education=traditional learning Skills: Blended digital education=traditional learning Patient-related outcome (ie, patients’ satisfaction): Traditional learning>blended digital education
	Gartmeier et al, 2015 [30] Germany, factorial RCT	168 (second and third)	IG2: 300-min online tutorial and role play (blended digital education) CG2: Role play	Skills: Likert scale	Narrative presentation	Skills: Blended digital education>traditional learning
	Lanken et al, 2015 [31] US, cluster RCT	370 (second and third)	IG: 1-h online module and small group discussion (blended digital education) CG: Traditional learning (usual curriculum)	Skills: survey Attitude: survey (Cronbach alpha=.89)	Skills: SMD=–0.08 (–0.28 to 0.13) Attitude (toward the outcome): SMD=0.05 (–0.15 to 0.26)	Skills: Blended digital education=traditional learning Attitude (toward the outcome): Blended digital education=traditional learning
	Lee et al, 2015 [32] US, mixed method RCT	119 (third)	IG: 1-h online module (cultural competency and PACT^i^ training plus standard curriculum) CG: Traditional learning (standard curriculum)	Knowledge: PACT (questionnaire) Skills: OSCE	Knowledge: SMD= 0.38 (0.02-0.74) Skills: SMD=0.05 (–0.31 to 0.41)	Knowledge: Blended digital education>traditional learning Skills: Blended digital education=traditional learning
	Ruesseler et al, 2017 [37] Germany, RCT	100 (fourth)	IG: 1.5-h video-assisted oral feedback (blended digital education: video recorded role play with video-assisted oral feedback) CG: Traditional learning (received direct oral feedback after role play )	Skills: OSCE	Skills: SMD=0.92 (0.51-1.33)	Skills: Blended digital education>traditional learning
	Tang et al, 2017 [39] China, RCT	95 (fourth)	IG: Online video plus team discussion (blended online digital education) CG: Traditional learning (didactic lecture)	Attitude (toward the outcomes): Likert scale (three-point, validated) Students’ satisfaction (with the intervention) Likert scale (three-point, validated)	Attitude (toward the outcomes): favored IG over CG (*P*=.04) Students’ satisfaction (with the intervention): no difference (*P*=.61)	Attitude (toward the outcomes): Blended online digital education>traditional learning Students’ satisfaction (with the intervention): Blended online digital education=traditional learning
**Digital education (more interactive) versus digital education (less interactive)**						
	Bearman et al, 2001 [28] Australia, RCT	284 (not specified)	IG: 1-h problem-solving VP CG: 1-h narrative VP	Skills: scale (Cronbach alpha=.83)	Skills: SMD= –0.12 (–0.43 to 0.2)	Skills: Digital education (more interactive)=digital education (less interactive)
	Chittenden et al, [29] US, RCT	121 (third)	IG1: 45-min online module (multimedia) IG2: 45-min online module (classic)	Skills: checklist, Likert scale	Skills: SMD=0.00 (–0.42 to 0.42)	Skills: Digital education (more interactive)=digital education (less interactive)
	Foster et al, 2015 [35] US, RCT	67 (second)	IG: Online-based VP simulation CG: Video group (online module)	Skills: communication checklist and rapport subscale (Cronbach alpha=.97 and .84, respectively) Students’ satisfaction: survey (Cronbach alpha=.89)	Skills: SMD=0.33 (–0.15 to 0.82) Students’ satisfaction: *P*=.007)^j^	Skills: Digital education (more interactive)=digital education (less interactive) Students’ satisfaction: Digital education (online-based video group)>digital education (online-based VP simulation)
	Kron et al, 2017 [38] US, mixed method cluster RCT	421 (second)	IG: two VP simulations CG: online module (multimedia computer-based learning)	Skills: OSCE Attitude: survey	Skills: SMD=0.26 (0.06-0.45) Attitude (toward the intervention): SMD=0.71 (0.51-0.91)	Skills: Digital education (more interactive)>digital education (less interactive) Attitude (toward the intervention): Digital education (more interactive)>digital education (less interactive)

^a^SMD: standardized mean difference.

^b^RCT: randomized controlled trial.

^c^IG: intervention group.

^d^CG: control group.

^e^VP: virtual patient.

^f^SP: standardized patient.

^g^OSCE: Objective Structured Clinical Examination.

^h^MCQ: multiple-choice questionnaire.

^i^PACT: Problem Affect Concern Treatment.

^j^Rating of the technology module overall.

**Figure 2:**
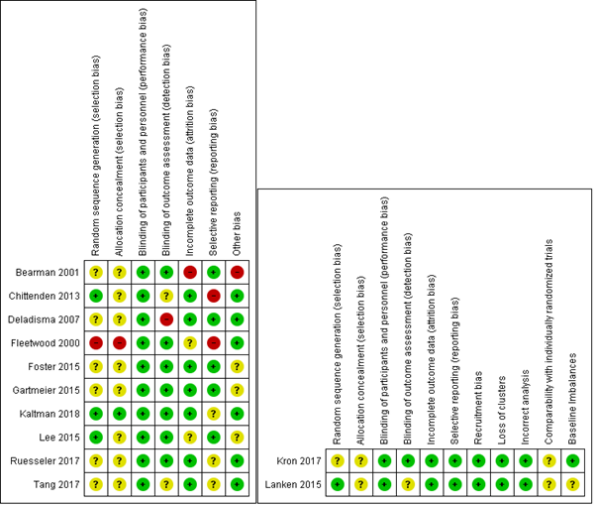
Risk of bias summary: review authors’ judgements about each risk of bias item for each included study.

In general, the risk of performance, detection, and attrition was predominantly low, and it was unclear or high for sequence generation bias, allocation concealment, and other bias. Reporting bias was judged as high in two (16.7%) of the studies. For two cRCTs, the overall risk of bias was low or unclear. Four of the 12 included studies (33.3%) were judged to have a high risk of bias in at least one domain (Figure 2). The quality of evidence ranged from moderate to very low due to study limitations, inconsistency, and imprecision across the studies.

### Effect of the Interventions

#### Digital Education Versus Traditional Learning

**Figure 3:**
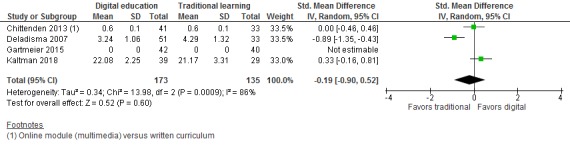
Forest plot of studies comparing digital education with traditional learning for postintervenion skill outcome, IV: inverse variance; random: random effect model.

Four studies [29,30,33,36] compared the effectiveness of digital education and traditional learning and reported on postintervention skills, attitudes, and satisfaction outcomes. For skills, there was no statistically significant difference between the digital education group (ie, online modules, tutorials, and virtual patient simulation) and the traditional learning group at postintervention (SMD=–0.19; 95% CI –0.9 to 0.52; 3 studies (304 students); *I*^2^=86%; low-quality evidence; Figure 3). However, this finding had high imprecision with wide CIs, which also included a large effect size in favor of traditional learning as well as a moderate effect size in favor of digital education. The high observed heterogeneity was largely driven by a study comparing the effectiveness of VP simulation to simulated patient training [33]. The remaining two studies compared the effectiveness of online modules or VP simulation with more passive forms of traditional learning such as written materials or usual curriculum [29,36]. Findings from one study [30] favoring online digital education over no intervention could not be pooled with the other studies due to the lack of comparable numerical data.

None of the studies reported on knowledge, attitudes, satisfaction, adverse effects, patient outcomes, or cost outcomes.

#### Blended Digital Education Versus Traditional Learning

**Figure 4:**
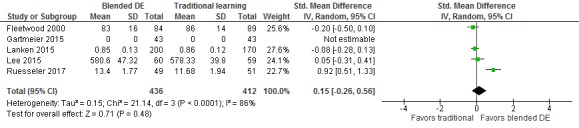
Forest plot of studies comparing blended digital education with traditional learning for postintervention skill outcome. IV: inverse variance; random: random effect model; DE: digital education.

Six studies [30-32,34,37,39] compared the effectiveness of blended digital education (ie, blended online or offline [video-based] digital education) and traditional learning and assessed students’ postintervention knowledge, skills, attitude, and patient-related outcomes (ie, patients’ satisfaction). For skills, there was no statistically significant difference between the groups at postintervention (SMD=0.15; 95% CI –0.26 to 0.56; *I*^2^=86%; 4 studies (762 students); small effect size; low-quality evidence; Figure 4). The reported findings were imprecise due to wide CIs including moderate effect sizes in favor of blended digital education. Three studies included in the meta-analysis compared a blend of online modules and a small group discussion or standard curriculum with standard curriculum or small group discussions only [31,32,34]. The high observed heterogeneity was largely driven by a study comparing role play and video-assisted oral feedback to role play with oral feedback only, favoring blended digital education [37]. Findings from one study favoring a blend of online tutorials and role play could not be included in the meta-analysis due to the lack of comparable outcome data [30].

**Figure 5:**
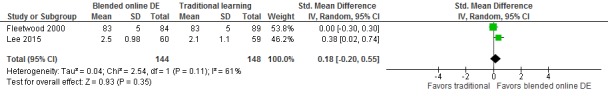
Forest plot of studies comparing blended online digital education with traditional learning for postintervenion knowledge outcome. IV: inverse variance; random: random effect model; DE: digital education.

For knowledge, two studies compared the effectiveness between blended online digital education and traditional learning and reported no statistically significant difference between the groups at postintervention (SMD=0.18; 95% CI –0.2 to 0.55; *I*^2^=61%; 292 students; low-quality evidence; Figure 5). Wide CIs around the pooled estimate also included moderate effect size in favor of blended online digital education.

Two studies [31,39] assessed students’ attitude toward the outcome (skills acquisition) at postintervention and reported no difference between the groups [31] or favored blended online education over traditional learning with didactic lectures (*P*=.04) [39].

One study also assessed students’ satisfaction with the intervention at postintervention and reported no difference between the groups (*P*=.61) [39]. One study [34] reported patient-related outcomes (ie, patients’ satisfaction) and compared a blend of online modules and small group discussions (ie, blended online digital education) with a control group of small group discussions only. The study reported slightly higher patients’ satisfaction scores in the control group than in the blended online digital education (SMD=–0.43; 95% CI –0.73 to –0.13). None of the studies reported on the adverse effects or cost outcomes.

#### Digital Education (More Interactive) Versus Digital Education (Less Interactive)

**Figure 6:**
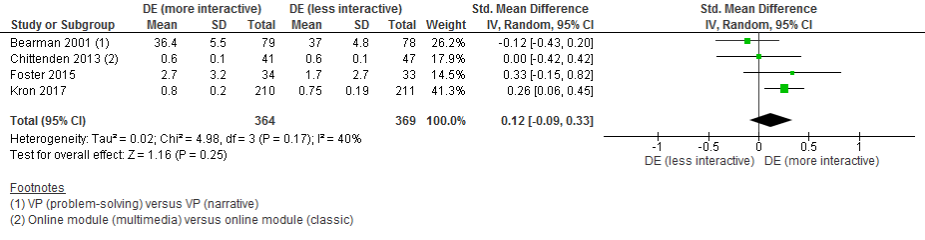
Forest plot of studies comparing digital education (more interactive) with digital education (less interactive) for postintervention skills outcome. IV: inverse variance; random: random effect model; DE: digital education.

Four studies [28,29,35,38] compared the effectiveness of more and less interactive digital education and assessed postintervention skills, attitudes, and satisfaction. More interactive forms of digital education (ie, problem solving, VP simulation, and online multimedia modules) reported similar effectiveness or no difference in postintervention skills compared to less interactive forms of digital education (ie, narrative virtual patient simulation, online video-based learning, and classic online modules) (SMD=0.12, 95% CI –0.09 to 0.33, *I*^2^=40%, 4 studies [893 students], moderate-quality evidence; Figure 6).

One study [38] assessed students’ attitude towards the intervention and reported moderate beneficial effect on postintervention attitude scores in the VP group compared to the online module group (SMD=0.71; 95% CI: 0.51-0.91). One study [35] assessed students’ satisfaction and reported that students were more satisfied with VP simulation than the online-based video module (*P*=.007). None of the studies reported on knowledge, adverse effects, patient outcomes, or cost outcomes.

## Discussion

### Principal Findings

This systematic review assessed the effectiveness of digital education on medical students’ communication skills compared to traditional learning or other forms of digital education. We summarized and critically evaluated evidence for the effectiveness of digital education for medical students’ communication skills training. Twelve studies with 2101 medical students met the eligibility criteria. We found low-quality evidence with wide CIs and high heterogeneity, showing no statistically significant difference between digital education and traditional learning in terms of communication skills. Blended digital education seems to be at least as effective as and potentially more effective than traditional learning for communication skills and knowledge. We also found no difference in postintervention skills between more and less interactive forms of digital education. Data on attitudes and satisfaction were limited and mixed. No study reported adverse or unintended effects of digital education nor conducted an economic evaluation. The majority of the studies (N=9, 75%) had a high risk of bias. The quality of evidence ranged from moderate to very low due to the study limitations, inconsistency, and indirectness (Multimedia Appendices 2-4).

The included studies differed considerably in terms of intervention, comparators, and outcome measures used, showing a wide scope of potential for the use of digital education in communication skills training for medical students. However, limited primary studies consisting of data with high heterogeneity prevent us from drawing strong conclusions on the topic. Furthermore, seven (58.3%) of the included studies failed to provide details of sample size or power calculations [28,29,32,35-37,39]. The included studies may have therefore been underpowered and unable to detect change in learning outcomes. Finally, the effect sizes were typically small. Other limitations pertained to the risk of bias. Overall, four of the 12 included studies (33.3%) were judged to have a high risk of bias in at least one domain.

The included evidence has some limitations. First, most of the studies were conducted in high-income countries (except one study that was conducted in China), which might further limit the transferability or applicability of the evaluated evidence in low- and middle-income countries. Second, the included studies focused only on specific forms of digital education such as online or offline digital education and VP simulation, and there is a need to explore the effectiveness of other forms of digital education such as virtual reality, serious gaming, mobile learning, and massive open online courses on the topic. Third, all included studies assessed short-term effectiveness of the interventions (ie, assessed effectiveness immediately after the intervention), and there is a need to assess long-term effectiveness of interventions through aspects such as knowledge retention and skills retention at 3-month or 6-month follow-ups. Lastly, the included studies mostly evaluated skills outcome, and there is limited evidence for other outcomes such as knowledge, attitude, satisfaction, adverse or untoward effects of the intervention, and patient and cost-related outcomes.

### Implications for Future Research

We identified the need for further, more methodologically sound research that may lead to more conclusive findings. Studies identified in this review have many significant methodological weaknesses, from inadequate power to unclear theoretical underpinnings; insufficient description of educational interventions (complexity, duration and intensity); uncertainty of what constitutes a change (compared with baseline); little, if any, description of technical features; skills retention (follow-up); and comparability of the content delivered digitally or traditionally. The use of validated and reliable measurement tools is paramount to advancing the field [40], as its transparent description on the level of trialists’ involvement in instructions, outcome(s) in the background, usability testing, and data protection policies could affect the results of the outcomes. Other important factors that need further research include the availability of infrastructure, financial incentives for learners, previous experience in digital education, barriers or facilitators, cost evaluation, fidelity, adverse effects, and access to power supply. Finally, incorporation of evidence from low- and middle-income countries should increase generalizability and applicability in those settings.

### Strengths and Limitations of the Review

Strengths of this study include comprehensive searches with no language limitations and robust screening, data extraction, risk of bias assessments, and a critical appraisal of the evidence. Nevertheless, some limitations must be acknowledged while interpreting the results of this systematic review. There was a considerable degree of methodological and clinical heterogeneity in pooled analyses, and the applicability of evaluated evidence might be limited due to high heterogeneity. Additionally, most of the included studies (92%) reported postintervention data, and we could not calculate pre-post intervention change scores. We also assumed that baseline characteristics including measure scores were adjusted before randomization. Finally, we were unable to obtain additional information from the study authors in six studies that reported mixed participants and mixed results.

### Conclusions

The findings from this review suggest that digital education (standalone or blended with traditional learning) could be as effective as traditional learning (ie, didactic lectures, groups discussions, role play, or oral feedback) in improving postintervention communication skills for medical students. Similarly, more interactive forms of digital education have similar effectiveness or skills outcome compared to less interactive forms of digital education in terms of participant’ skills. The overall risk of bias was high, and the quality of evidence ranged from moderate to very low for the reported outcomes. There is a need for further research assessing long-term effectiveness including knowledge or skills retention, other outcomes such as patient-related outcomes, and cost-effectiveness as well as other forms of digital education for medical students’ communication skills training.

## Appendix

Multimedia Appendix 1 Medline (Ovid) Search Strategy.

Multimedia Appendix 2 Summary of the findings table for the effects of digital health education on communication skills.

Multimedia Appendix 3 Summary of findings table for the effects of blended digital health education on communication skills.

Multimedia Appendix 4 Summary of findings table for the effects of digital education (more interactive) compared to digital education (less interactive) on communication skills.

## Abbreviations

CG: control group
cRCT: cluster randomized controlled trial
GRADE: Grading of Recommendations, Assessment, Development and Evaluations
IG: intervention group
MCQ: multiple-choice questionnaire
OSCE: Objective Structured Clinical Examination
PACT: Problem Affect Concern Treatment
PRISMA: Preferred Reporting Items for Systematic Reviews and Meta-Analyses
RCT: randomized controlled trial
SMD: standardized mean difference
SP: standardized patient
VP: virtual patient
