# Supplementary material for: Effectiveness of Digital Education on Communication Skills Among Medical Students: Systematic Review and Meta-Analysis by the Digital Health Education Collaboration
Source: J Med Internet Res. 2019 Aug 27;21(8):e12967. doi: 10.2196/12967 (PMC6764329; doi:10.2196/12967)
Supplement: Multimedia Appendix 2 [file jmir_v21i8e12967_app2.pdf]

Multimedia Appendix 2. Summary of Findings Table for the Effects of Digital Health Education on communication skills

**Digital education (online learning, virtual patient) compared with traditional**				
**Patient or population**: first, second, third and/or fourth year undergraduate medical students<br>**Settings**: Universities<br>**Intervention**: Digital education (online digital education, VP simulation)<br>**Comparison**: Traditional learning				
**Outcomes**	**Illustrative comparative risks (95% CI)**	**Number of participants (number of studies)**	**Quality of the evidence (GRADE)**	**Comments**
**Skills** (measured with surveys, checklist, Likert scales, OSCE)	The mean skills score in digital education groups was -0.19 standard deviations lower (-0.9 lower to 0.52 higher).	472 students<br>(4 studies)	⊕⊕⊖⊖<br>low [a,b]	The standard deviations was derived from a SMD of -0.19 (95% CI: -0.9 to 0.52) which indicates a small effect size. The result of one study (168 participants) was not added to the meta-analysis due to incomplete outcome data. However, the study authors reported improved effectiveness in postintervention skills with online digital education compared to traditional learning [30].
**Knowledge**	No studies reported knowledge outcome.			

**Attitude**	No studies reported attitude outcome.
**Satisfaction**	No studies reported satisfaction outcome.
**Patient-related outcome**	No studies reported patient-related outcome.
**Adverse outcome**	No studies reported adverse events.
**Economic evaluation**	No studies reported economic evaluation.
OSCE-objective structured clinical examination; VP- virtual patient.<br><br>GRADE Working Group grades of evidence<br>**High quality**: further research is very unlikely to change our confidence in the estimate of effect.<br>**Moderate quality**: further research is likely to have an important impact on our confidence in the estimate of effect and may change the estimate.<br>**Low quality**: further research is very likely to have an important impact on our confidence in the estimate of effect and is likely to change the estimate.<br>**Very low quality**: we are very uncertain about the estimate.	

Table 2 Legend:

[a] Downgraded by one level for study limitations: the risk of bias was unclear or high in most included studies.

[b] Downgraded by one level for inconsistency: the heterogeneity between studies was high with a lack of overlap among confidence intervals.
