# Supplementary material for: Effectiveness of Digital Education on Communication Skills Among Medical Students: Systematic Review and Meta-Analysis by the Digital Health Education Collaboration
Source: J Med Internet Res. 2019 Aug 27;21(8):e12967. doi: 10.2196/12967 (PMC6764329; doi:10.2196/12967)
Supplement: Multimedia Appendix 3 [file jmir_v21i8e12967_app3.pdf]

Multimedia Appendix 3. Summary of Findings Table for the Effects of Blended Digital Health Education on communication skills

Digital education (online learning, virtual patient) compared with traditional				
**Patient or population**: second and/or third or fourth year undergraduate medical students<br>**Settings**: Universities<br>**Intervention**: Blended digital education (online or offline digital education plus traditional learning)<br>**Comparison**: Traditional learning (i.e. didactic lecture, group discussions, role-play, oral feedback, usual or standard curriculum)				
**Outcomes**	**Illustrative comparative risks (95% CI)**	**Number of participants (number of studies)**	**Quality of the evidence (GRADE)**	**Comments**
**Knowledge** (measured with MCQ, questionnaire) (postintervention)	The mean knowledge score in blended online digital education groups was 0.18 higher (-0.20 lower to 0.55 higher)	292 students (2 studies)	⊕⊕⊖⊖ low [a,b]	The standard deviations was derived from a SMD of 0.18 (95% CI: -0.2 to 0.55) which indicates a small effect size. Two studies assessed the effectiveness of blended online digital education compared to traditional learning with standard curriculum or group discussions and reported no difference in postintervention knowledge scores between the groups [32, 34].
**Skills** (measured with surveys, checklist, Likert scales, OSCE) (postintervention)	The mean skills score in blended digital education groups was 0.15 higher (-0.26 lower to 0.56 higher).	762 students (4 studies)	⊕⊕⊖⊖ low [a,b]	The standard deviations was derived from a SMD of 0.15 (95% CI: -0.26 to 0.56) which indicates a small effect size. The result of one study (168 participants) was not added to the meta-analysis due to incomplete outcome data. However, the study authors reported improved

				effectiveness with online digital education in postintervention skills compared to traditional learning [30].
**Attitude** (measured with three-point Likert scale, survey) (postintervention)	Not estimable	446 students (2 studies)	⊕⊕⊖⊖ low [a,b]	One study [39] reported higher postintervention attitude scores in blended online education compared to traditional learning with didactic lectures ($P = .037$) and one study [31] reported no difference in postintervention attitude scores between the groups.
**Satisfaction** (measured with three-point Likert scale, validated checklist) (postintervention)	Not estimable	76 students (1 study)	⊕⊕⊖⊖ low [a,b,c]	One study [39] assessed students' satisfaction with the intervention and reported no difference between the groups ($P = .610$).
**Patient-related outcomes** (measured with validated checklist)	Not estimable	173 students (1 study)	⊕⊕⊖⊖ low [a,b]	One study [34] assessed patients' satisfaction with blended online digital education and reported that patients' satisfaction were higher in traditional learning group compared to the intervention (SMD=-0.43, 95% CI: -0.73 to -0.13).
**Adverse outcome**	No studies reported adverse events.			
**Economic evaluation**	No studies reported economic evaluation.			

OSCE-objective structured clinical examination; VP- virtual patient.<br>GRADE Working Group grades of evidence<br>**High quality**: further research is very unlikely to change our confidence in the estimate of effect.<br>**Moderate quality**: further research is likely to have an important impact on our confidence in the estimate of effect and may change the estimate.<br>**Low quality**: further research is very likely to have an important impact on our confidence in the estimate of effect and is likely to change the estimate.<br>**Very low quality**: we are very uncertain about the estimate.

Table 3 Legend:

[a] Downgraded by one level for study limitations: the risk of bias was unclear or high in most included studies.

[b] Downgraded by one level for inconsistency: the heterogeneity between studies was high.

[c] Rated down by one level for imprecision: number of participants (effective sample size) in the study is less than the number of patients generated by a conventional sample size calculation for a single adequately powered trial (optimal information size)
