# Supplementary material for: Effectiveness of Digital Education on Communication Skills Among Medical Students: Systematic Review and Meta-Analysis by the Digital Health Education Collaboration
Source: J Med Internet Res. 2019 Aug 27;21(8):e12967. doi: 10.2196/12967 (PMC6764329; doi:10.2196/12967)
Supplement: Multimedia Appendix 4 [file jmir_v21i8e12967_app4.pdf]

Multimedia Appendix 4. Summary of Findings Table for the Effects of Digital Education (more interactive) compared to Digital Education (less interactive) on communication skills

**Digital education (more interactive) vs digital education (less interactive)**				
**Patient or population**: second or third year undergraduate medical students<br>**Settings**: Universities<br>**Intervention**: digital education (more interactive forms: VP simulations and online multimedia modules)<br>**Comparison**: digital education (less interactive forms: online classic modules, problem solving VP)				
**Outcomes**	**Illustrative comparative risks (95% CI)**	**Number of participants (number of studies)**	**Quality of the evidence (GRADE)**	**Comments**
**Skills** (measured with checklist, Likert scales, OSCE) (postintervention)	The mean skills score in blended digital education groups was 0.12 higher (-0.09 lower to 0.33 higher).	864 students<br>(4 studies)	⊕⊕⊕⊖<br>moderate [a]	The standard deviations was derived from a SMD of 0.12 (95% CI: -0.09 to 0.33) which indicates no difference between the two groups.
**Attitude** (measured with survey) (postintervention)	Not estimable	421 students<br>(1 study)	⊕⊕⊖⊖<br>low [a,b]	One study [38] assessed students' attitude towards the intervention and reported moderate improvement in postintervention attitude scores with VP simulation compared to online

				module (SMD=0.71, 95% CI: 0.51 to 0.91).
**Satisfaction** (measured with survey) (postintervention)	Not estimable	67 students<br>(2 studies)	⊕⊖⊖⊖<br>very low [a,b,c]	One study [35] assessed students' satisfaction and reported that students' were more satisfied with online-based VP simulation compared to online-based video module (P= .007).
**Knowledge**	No studies reported knowledge outcome.			
**Patient-related outcome**	No studies reported patient-related outcome.			
**Adverse outcome**	No studies reported adverse events.			
**Economic evaluation**	No studies reported economic evaluation.			
OSCE-objective structured clinical examination; VP- virtual patient.<br><br>GRADE Working Group grades of evidence<br>**High quality**: further research is very unlikely to change our confidence in the estimate of effect.<br>**Moderate quality**: further research is likely to have an important impact on our confidence in the estimate of effect and may change the estimate.<br>**Low quality**: further research is very likely to have an important impact on our confidence in the estimate of effect and is likely to change the estimate.<br>**Very low quality**: we are very uncertain about the estimate.				

Table 4 Legend:

[a] Downgraded by one level for study limitations: the risk of bias was unclear or high in most included studies.

[b] Rated down by one level for inconsistency: the heterogeneity is high with large variations in effect and lack of overlap among confidence intervals (CIs).

[c] Rated down by one level for imprecision: number of participants (effective sample size) in the study is less than the number of patients generated by a conventional sample size calculation for a single adequately powered trial (optimal information size)
